# Tasked-Based Functional Brain Connectivity in Multisensory Control of Wrist Movement After Stroke

**DOI:** 10.3389/fneur.2019.00609

**Published:** 2019-06-13

**Authors:** Benjamin T. Kalinosky, Kaleb Vinehout, Miguel R. Sotelo, Allison S. Hyngstrom, Brian D. Schmit

**Affiliations:** ^1^Integrative Neural Engineering and Rehabilitation Laboratory, Department of Biomedical Engineering, Marquette University and the Medical College of Wisconsin, Milwaukee, WI, United States; ^2^Integrative Neural Engineering and Rehabilitation Laboratory, Department of Physical Therapy, Marquette University, Milwaukee, WI, United States

**Keywords:** stroke, functional connectivity, upper limb, sensory integration, task based approach

## Abstract

In this study we documented brain connectivity associated with multisensory integration during wrist control in healthy young adults, aged matched controls and stroke survivors. A novel functional MRI task paradigm involving wrist movement was developed to gain insight into the effects of multimodal sensory feedback on brain functional networks in stroke participants. This paradigm consisted of an intermittent position search task using the wrist during fMRI signal acquisition with visual and auditory feedback of proximity to a target position. We enrolled 12 young adults, 10 participants with chronic post-stroke hemiparesis, and nine age-matched controls. Activation maps were obtained, and functional connectivity networks were calculated using an independent component analysis (ICA) approach. Task-based networks were identified using activation maps, and nodes were obtained from the ICA components. These nodes were subsequently used for connectivity analyses. Stroke participants demonstrated significantly greater contralesional activation than controls during the visual feedback condition and less ipsilesional activity than controls during the auditory feedback condition. The sensorimotor component obtained from the ICA differed between rest and task for control and stroke participants: task-related lateralization to the contralateral cortex was observed in controls, but not in stroke participants. Connectivity analyses between the lesioned sensorimotor cortex and the contralesional cerebellum demonstrated decreased functional connectivity in stroke participants (*p* < 0.005), which was positively correlated the Box and Blocks arm function test (*r*^2^ = 0.59). These results suggest that task-based functional connectivity provides detail on changes in brain networks in stroke survivors. The data also highlight the importance of cerebellar connections for recovery of arm function after stroke.

## Introduction

In this study, we used functional magnetic resonance imaging (fMRI) and a novel task paradigm to investigate the effects of multimodal sensory feedback on detection of brain functional networks after stroke. In prior studies, the primary motor cortical regions and their pathways have been a major focus in investigating the functional effects of stroke lesions on the brain (1–4). However, brain lesions might have an even stronger impact on integrative networks that process multisensory inputs and plan movements in a functional context. Lesions affecting sensorimotor integrative networks of the brain are likely to play a critical role in recovery, and damage to these networks could lead to chronic impairment, as they are important to motor learning and recovery (2, 4–9). Although current measures of functional connectivity characterize communication between brain motor regions, the changes in connectivity of the sensorimotor association areas have been largely unexplored in stroke survivors. In order to characterize the function of sensorimotor networks in stroke survivors, we measured brain connectivity and activation with functional magnetic resonance imaging (fMRI) during rest and during tasks that invoked key features of sensorimotor and multisensory integration.

fMRI has been used to characterize differences in brain activation patterns in stroke survivors and to document cortical plasticity with natural recovery or following targeted therapeutic interventions. In general, increased intensity and spread of brain activation after stroke have been associated with decreased functional outcomes. During finger movement, stroke survivors have increased cortical activation with a broader spatial extent in the ipsilesional hemisphere as well as contralesional activity that is absent in controls (10). Increases in cortical activity distant from primary motor areas impacted by a stroke lesion are considered evidence for cortical reorganization (11). These plastic changes might be compensatory or conversely, they could be maladaptive for functional recovery. During recovery, the intensity, and spread of brain activation associated with hand grip decreases in lower functioning stroke survivors (4), suggesting that simple task-related brain activation volumes are inversely correlated with functional recovery. In contrast, decreased activity in cortical areas have been documented in stroke survivors using the relatively more complex task of bilateral pedaling during fMRI (12), possibly due to a greater reliance on integrative sensorimotor regions that might become dysfunctional after stroke. The recent emergence of brain functional connectivity analyses (13, 14) offers the opportunity to interpret changes in task-related brain activity in the context of brain networks, potentially offering insight into the mechanisms underlying changes in brain function after stroke.

Functional connectivity analyses also provide evidence of changes in brain function after stroke. Functional connectivity MRI (fcMRI) (13, 15) infers coactivation of one or more cortical areas by their correlated fMRI signal over time. This analysis can be used to identify functional networks using fMRI signals obtained at rest (13) or during a task (15). For this analysis, nodes can be defined using predetermined anatomical regions of interest or identified by measuring regional homogeneity of voxel-wise intrinsic functional connectivity (16). Alternatively, functional connectivity can be decomposed into a set of spatiotemporal networks using an independent component analysis (17, 18); an independent component consists of a 3D volume that provides each voxel's contribution to a network and a BOLD time-course that is shared by all voxels within that network. In resting-state fMRI, one of the most consistent findings in stroke participants is decreased functional connectivity between the ipsilesional and contralesional sensorimotor cortices (19–21). While resting state functional connectivity and task-based connectivity share correspondence, they also have many notable differences (22–24). In healthy individuals, the spatial extent of nodes determined by an independent component analysis is similar across task and resting state paradigms (25). In contrast, task-based connectivity exhibits “local specialization” with increased connectivity of long-distance connections compared to resting state analyses (26, 27). This means that areas engaged in a given task have increased local connectivity in the task-specific area (i.e., local specialization) and increased global connectivity (long distance connections) between the different areas engaged with that task. For a multimodal sensorimotor task, we expect increased local specialization within the primary sensorimotor region and increased long-distance communication between the ipsilesional sensorimotor cortices and contralesional cerebellum, similar to activation results reported during complex motor tasks (28).

Functional connectivity analyses are particularly effective in quantifying functionally relevant changes in brain networks after stroke and during recovery (29). It has been suggested that network integrity and reorganization is critical for functional recovery after stroke (30). Each cortical region can actively participate in multiple functional networks, allowing the brain to reorganize after damage to a particular node. Network plasticity has been documented in the motor network of people with stroke (31) and the integrity of contralesional parietofrontal and sensorimotor cortical networks has been associated with lower motor impairment after stroke (6). These findings suggest that resting state and task-based connectivity of sensorimotor integration areas predict motor function, and plasticity of these networks provide mechanisms for restoring motor function.

In order to identify changes in sensorimotor networks in chronic stroke survivors, we calculated the functional connectivity of the brain using resting state and task-based MRI, with a unique sensorimotor task that employs sensorimotor and multisensory integration. The task was specifically designed to engage integrative sensorimotor networks during controlled wrist movement. We then compared the changes in these networks to a clinical measure of upper limb function. We hypothesized that only during the task, functional connectivity between brain networks associated with sensorimotor integration would be reduced in stroke survivors, and that the reduction would be correlated to arm function.

## Methods

### Data Collection

#### Participant Recruitment and Clinical Testing

Twelve young adults (four female, 25.2 ± 2.4 years), 10 individuals with chronic post-stroke hemiparesis (four female, age 66.7 ± 7.94 years, at least 1.1 years since stroke), and nine age-matched control participants (five female, age 64.2 ± 7.73 years) participated in this study. The young adults were included as additional data to validate the new task paradigm in its ability to show areas of the brain involved in performing the task. Each participant provided informed written consent to the experimental protocol, which was approved by the Institutional Review Boards at Marquette University and the Medical College of Wisconsin. Inclusion criteria included a history of an ischemic cortical or subcortical stroke that occurred no <6 months prior to recruitment. Participants with no ability to perform supination, pronation, ulnar deviation, or radial deviation of the wrist were excluded. Controls without history of stroke or other neurological impairments were age-matched (within 3 years) and gendered-matched to the stroke participants. Each stroke participant completed the upper extremity (UE) portion of the Fugl-Meyer Assessment (32) for a maximum possible score of 126. Participants also completed the Box and Blocks Test of Manual Dexterity (33), the Wolf Motor Function Test (34) for upper extremity motor ability (maximum score of 75), and the Modified Ashworth Scale (35). These clinical measurements were correlated with measured brain activity during the task and the functional connectivity during resting state and during the task. The stroke participant clinical scores and lesion locations are shown in Table 1, and Figure S1 shows the lesion distribution; although there is little lesion overlap, the lesion distribution shows damage in the sensorimotor pathways.

**Table 1:** Stroke participant clinical scores and lesion location.

**Subject ID**	**S04**	**S05**	**S07**	**S08**	**S10**	**S12**	**S14**	**S15**	**S16**	**S18**
More affected side	Left	Right	Right	Left	Right	Left	Left	Right	Right	Right
Dominant side	Right	Right	Right	Right	Right	Right	Right	Right	Right	Right
Lesion location	Right insula, right temporal lobe	Left PLIC (CST)	Left pons (CST)	Right PLIC, Left PLIC (anterior to CST)	Left superior parietal	Right subcortical/cortical, PrCG, PoCG	Left cerebellum	Left PLIC (CST)	Unknown, possibly left CP	Left brainstem, left PLIC
Box and blocks	29	64	47	41	66	64	38	56	57	62
Wolf motor (/75)	71	72	38	72	75	54	58	74	75	74
Fugl-Meyer upper extremity (/126)	124	106	82	122	124	97	120	126	124	122

*Participant information including affected and dominant limb, lesion location, and clinical scores including Box and Blocks, Wolf Motor, and Fugl-Meyer upper extremity (CST, corticospinal tract; PLIC, posterior limb of the internal capsule; PrCG, pre-central gyrus; PoCG, post-central gyrus; CP, cerebral peduncle)*.

#### Experimental Paradigm

Our sensorimotor integration experiment was designed with cues and feedback that contrasted the effects of auditory and visual sensation. We introduced a task paradigm for studying the role of sensory integration in complex movement. Chronic stroke participants have more difficulty coordinating sensorimotor behavior, especially in tasks with higher complexity (36). Our task required the participant to produce movement while integrating multiple sensory modalities.

##### Motion recording and audiovisual feedback

**Figure 1:**
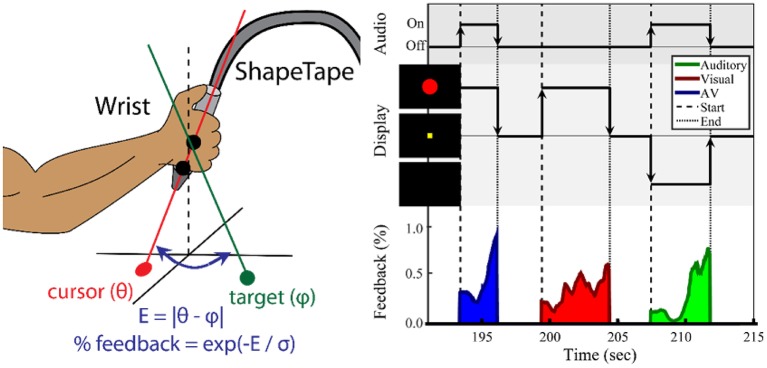
Task paradigm for wrist movement and sensorimotor integration. **(Left)** Illustration of the ShapeTape apparatus. The forearm position was fixed. **(Right)** Experimental design of the multisensory search task. During each trial, the participant maximized sensory feedback using wrist joint angles to minimize error to a target angle. The participant was instructed to move the wrist to maximize the circle diameter and/or a sound volume. Auditory, visual, or audiovisual feedback were presented at the start and during each trial. After reaching the target, the participant fixated on a yellow square during a 2–4 s intertrial period. The average trial duration was 5 s.

Every participant completed two sessions on separate days no more than 2 weeks apart. Participants were trained to perform a wrist-movement task during the first session. The second session used the same wrist movement task and consisted of a second training period 1 h before the task-based fMRI session. The experimental apparatus is shown in Figure 1 (left). The forearm of the impaired limb was fixed on a small ramp to allow for radial and ulnar deviation of the wrist. Both the elbow and the forearm were secured on the ramp to ensure that the task was controlled by wrist movement only. The hand gripped the end of a ShapeTape device (Measurand Inc., Canada), an array of 16 optical fiber sensor pairs that provide 3D Euclidean coordinates along the sensor region. Each sensor pair also provided a 3D rotation matrix expressed in quaternion form. Visual feedback was presented on a computer monitor, and speakers provided auditory feedback. Motion data were recorded every 24 ms, or 41.67 Hz.

Shown in Figure 1 (left), two sensor pairs at the end of the ShapeTape were used to calculate a 3D ray with angle **θ=**{θ_*x*_, θ_*z*_} relative to the horizontal (x-z) plane, which was used to define the orientation of the wrist. Effectively, pronation/supination was mapped to an angle within the x-y plane θ_*z*_, and radial and ulnar deviation was mapped to an angle in the z-y plane. Letting **p**_*n*_ and **p**_*n*−1_ be the 3D coordinates of the last two sensors, an orientation vector ***v*** was calculated as:

(1) $$\begin{array}{r} v=\frac{{\text{p}}_{n}-{\text{p}}_{n-1}}{\left\|{\text{p}}_{n}-{\text{p}}_{n-1}\right\|} \end{array}$$

The wrist orientation was estimated as θ_*x*_ = (*v*_*X*_/*v*_*Y*_), and θ_*Z*_ = (*v*_*Z*_/*v*_*Y*_). For each search task, a target angle, **φ**, was created. The error to **φ** during the search task was calculated as $E=\sqrt{{\left({\;\theta }_{X}-{\;\varphi }_{X}\right)}^{2}+{\left({\;\theta }_{Z}-{\;\varphi }_{Z}\right)}^{2}}$. *E* was then used to provide feedback to the participant related to wrist proximity to the target. The feedback intensity, *w*, was calculated as *w* = exp(−*E*/σ), with the sensitivity parameter σ fixed at 0.1 radians for all trials. Intensity *w* was used to modulate visual and auditory feedback stimuli. Auditory feedback was a 440 Hz tone and its volume was modulated linearly by *w*. Visual feedback was presented as a solid red circle at the center of a black screen. The circle diameter was modulated linearly by *w* from 15 to 160 pixels or 0.38–4.06 cm. The screen was placed ~2 feet away from the eyes. The resulting aperture angle was effectively varied 0.179–1.91 degrees.

##### Experimental design

As shown in Figure 1 (right), a search-based wrist movement task was designed to invoke brain networks involved in sensorimotor and multisensory integration. The first session began with up to five familiarization trials, in which the participant used the wrist to move a white cursor to a yellow square target at the center of the screen. These trials were also used to verify the participant's range of motion. Once comfortable reaching the square, the participant was then informed that the square and cursor would not be visible. At the start of each trial, one of three types of sensory feedback was presented. For both the training session and the fMRI session, a series of two search-task runs (“search”) (6 min each) and one sensory-motor (“SM”) only run (6 min) were conducted.

The “search” task run consisted of a series of trials, each trial including visual, auditory, or combined audiovisual feedback. In the visual-only feedback condition, a solid red circle appeared and grew larger as error decreased. Once the participant reached the target, the solid red circle was changed into an outline and then disappeared. During auditory-only trials, the participant searched for the target with feedback provided by the tone volume. Upon reaching the target, the tone was altered to a fixed pitch of 880 Hz, giving a “beep” sound, and turned off. In audiovisual feedback trials, the red circle and auditory tone were mapped independently to the x-coordinate and y-coordinate errors. The goal was to maximize both feedback intensities. After the target was reached, the participant fixated at a yellow square at the center of the screen for an inter-trial period with a normal random duration of 2.5 ± 0.5 s. Participants were notified that the trial would end automatically after an unspecified time if they failed to reach the target. A maximum trial duration of 15 s was used for all experiments.

A control task (“SM”) involving isolated sensory and motor tasks was conducted after the two search-based task runs. In the motor task, the words “Keep Moving” appeared on the screen, and the participant was told to move the wrist randomly in a similar pattern as during the search task. The participant was instructed to stop moving once the words disappeared. The message “Relax” was displayed on the screen for 2 s prior to the sensory only trials. For this condition, the participant was warned that there would be times during which the red circle and sound would appear and change outside of the participant's control. The participant was trained to not move and just watch and listen. The last instruction to the participant was “If you see the words “Keep moving” then move. If you do not see the words “Keep moving,” then stay still no matter what happens.” Throughout this final run, the experiment would alternate between motor-only and sensory-only conditions every 12 ± 2.0 s.

#### MRI Scans

Every participant was screened for MRI safety before entering the magnetic environment. An axial T1-weighted anatomical image was acquired using a fast spoiled gradient recalled (SPGR) pulse sequence, with TE: 3.2 ms, TR: 8.16 ms, flip angle: 12 deg, prep time: 450, bandwidth: 22.73, FOV: 240 mm, 156 1 mm slices, matrix size: 256 ×240. For functional MRI, a sagittal view gradient-echo echo-planar sequence was acquired with TE: 25 ms, TR: 2,000 ms, flip angle: 77 deg, FOV: 240 ×240 mm, 41 slices with 3.5 mm thickness. Four 6 min fMRI scans were performed, one for resting state, two for the search task, and one for the sensory motor only task.

#### MRI Experimental Setup

As the participant lay supine, the forearm was elevated with foam and fixed in place with sandbags. The ShapeTape was placed into the participant's impaired hand, or right hand in healthy adults. Visual feedback was projected to a visor attached to the head coil, and earbuds were placed into the ears to provide auditory feedback. The MRI scan session consisted of one resting-state run followed by three task-based runs. During the 6 min resting-state run, each participant was asked to close their eyes and stay alert. After the resting-state scan, the ShapeTape was placed into the participant's hand. If the participant had difficulty gripping the device, then a surgical wrap was used to keep the hand closed. The participant completed three 6-min runs of the same experiment conducted for the first session, including two search-based and one sensory-motor only.

### Image Registration and Lesion Side Normalization

Intersubject and intermodality image registration was completed in both healthy adults and stroke participants using fully automated techniques. Each participant's anatomical T1-weighted MRI volume was registered to a 152-brain MNI space using a 12-parameter affine registration, and then non-linear image registration was performed using Maxwell's demons algorithm (37). Local histogram matching was performed prior to deformable image registration in order to mitigate errors caused by lesion contrasts (38). In brief, each voxel in the subject's T1-weighted image was assigned a value from 0 to 1 based on its percentile for a 5 ×5 ×5 voxel neighborhood centered at that voxel. The resulting deformed T1-weighted image was histogram-matched to the MNI template, where the local percentile of the subject's T1-weighted image was matched to the same percentile of the MNI template. Finally, 5 iterations of the demons algorithm were performed at the full 1 mm resolution to align the edges of the images.

The images of all stroke participants that completed the experiment with the left arm were flipped over the sagittal plane so that all lesions were on the left side of MNI space. One of these participants had a lesion within the left cerebellum, which was flipped to the right side. The flipping placed all strokes outside the cerebellum on the left side of MNI space.

### fMRI Data Processing

#### General Linear Model for the Search Task

Task-based functional MRI analysis was performed with AFNI (Analysis of Functional NeuroImages, RRID:SCR_005927, afni.nimh.nih.gov/afni). Data were temporally resampled in order to correct for non-uniform slice acquisition timing within each volume. BOLD signal changes related to head translation and rotation were corrected by affine coregistration between volumes using AFNI's 3dvolreg function. The data were high-pass filtered above 0.01 Hz. The motion parameters included roll, yaw, pitch, and x, y, z translations, and were treated as coregressors for all subsequent analyses.

#### General Linear Model and Cortical Activation Maps

We were interested in stroke-related differences in cortical activity involved in sensorimotor integration during movement. In addition, we are interested in the young and age-matched control differences during this task. As previously shown in Figure 1, at the start of the trial the sensory feedback was at its lowest value. On the contrary, the level of wrist motion (not displayed in the figure) was greatest at the start of the search. As the participant closed in on the target, sensory feedback increased. In this latter phase of the task, finer wrist movements were required. Thus, wrist movement was greatest at the start of each trial, and sensory feedback was greatest at the end. In order to identify the brain activity associated with movement, wrist motion was estimated as the absolute change in **θ** with time. However, as mentioned above, the wrist motion parameters throughout time were used as co-regressors to help control for the variation in movement during the earlier and later stages of the task.

The sensory feedback and wrist movement signals, both produced with our in-house software from the ShapeTape data, were median filtered with a window of 2 s. Using the “waver” function in AFNI, the signals were then convolved with a double-gamma variate hemodynamic response function to produce modeled BOLD responses and resampled to the fMRI temporal resolution of 0.5 Hz. This method was repeated for the auditory, visual, and audiovisual feedback conditions to produce three movement regressors (A_M_, V_M_, AV_M_) and three sensory feedback regressors (A_S_, V_S_, AV_S_). Using the 3dDeconvolve program in AFNI, a multilinear regression was performed for each voxel, with the six task-related regressors and six head motion parameters (three rotation, three translation) contributing to the BOLD signal. The marginal *t*-value for the beta coefficient of each task-related regressor was resampled into 1 mm MNI space. These six cortical activation maps were calculated for every stroke participant and age-matched control.

#### Within Network Functional Connectivity

We were interested in the within network and between network functional connectivity. For the within network functional connectivity, functional connectivity MRI analysis was performed with Multivariate Exploratory Linear Decomposition into Independent Components (MELODIC) Version 3.14 available with the FMRIB's Software Library (FSL, RRID:SCR_002823, www.fmrib.ox.ac.uk/fsl). All runs and the stroke and aged matched control participants were time concatenated for a single 75-run (19 participants and 4 runs) group ICA. The sensory-motor only task run of stroke participant S04 was not included in the analysis since data collection was not complete. The data were high-pass filtered with a cutoff frequency of 0.01 Hz (39). The first five TRs were discarded to exclude signal drifts due to system ramp-up. This left each run with 175 volumes over 350 s. The functional image volumes were motion corrected using the MCFLIRT implementation (40). Slice-time correction was applied using linear interpolation. Skull-stripping was automatically performed with the brain extraction tool (BET) (41), and the data were spatially smoothed with a 5 mm full-width half-max Gaussian kernel. The skull stripped images were visually inspected to ensure the quality of the skull stripping. The resulting brain mask was used to exclude non-brain voxels from the remaining analysis. All participants were spatially normalized to an anatomical MNI standard template using a 12-parameter affine registration implemented in FLIRT (42). The voxel BOLD times series were demeaned, variance normalized, and whitened.

The number of independent components were estimated using a Bayesian approach described by Minka et al. (18). Once the independent components for the combined stroke and age matched control data were calculated, a dual regression (43) was used to estimate individual spatial maps and time courses for each participant and session. Components with vertical stripes in the axial view were associated with motion and excluded from further analysis. Voxel-based general linear modeling was performed between stroke and age-matched controls, and between rest and task conditions for each independent component. The resulting maps of t values were used as representations of the contributions of each condition to the component of interest. The term “within network functional connectivity” was used to refer to this relationship between the group/task and the network component.

#### Task-Based Network Identification

Overall relationships between resting-state networks and the task conditions were estimated with temporal correlation. The BOLD response to each task condition was modeled by convolving the stimulus presentation time signal with a double-gamma hemodynamic response function. The modeled time courses were concatenated across all 19 participants and four runs using the same arrangement used for the time-concatenated group ICA. The relationship between an independent component and the experimental variable was estimated by correlating the spatial component's data time course to the modeled BOLD response of the experimental variable. Since the movement-only and sensory-only conditions were presented in regular 15 s intervals, their modeled time courses were used to identify the functional brain network associated with the task. Shown in Figure S2, one particular task-related independent component (SM_L_) had a high correlation (*r* > 0.9) with the movement-only condition. This component, shown in Figure S2 below, will be referred to as the active sensorimotor network.

#### Between-Network Functional Connectivity Analysis

In order to assess the between-network functional connectivity, group ICA network maps were thresholded at a z-score of 30, and local maxima of clusters >2 cm^3^ were treated as nodes for a subsequent seed-based functional connectivity analysis. The z-score threshold and cluster size threshold were chosen heuristically such that only one or two clusters remained for each network. The voxel with the maximum z-score was used from each cluster for seed-based analysis. Shown in Table 2, a total of 27 local maxima were extracted from the left and right sensorimotor networks, the left and right parietofrontal control networks, the default-mode network, the bilateral cerebellar network, the bilateral extrastriate visual network, the primary visual network, the left and right auditory networks, and the bilateral thalamic network. Since the task-fMRI data were also included in the ICA, the left sensorimotor network map contained three clusters. These included the left precentral gyrus, and two clusters within the right cerebellum. The inclusion of task-positive BOLD data also caused the right sensorimotor network's local maximum to occur within the postcentral gyrus. Since the contralesional sensorimotor network has been shown to be involved in motor plasticity after stroke, the nodes from left and right sensorimotor networks were reflected over the mid-sagittal plane to produce 4 additional nodes. Independent components related to motion or cardiac artifact were regressed out of the raw BOLD data. The six motion regressors that were calculated by the MCFLIRT function prior to the group ICA were also used to clean the original BOLD data. The temporal correlation coefficient was calculated from the cleaned BOLD time courses of each pair of seed points.

**Table 2:** Locations used for between network seed-based FC analysis.

**Network**	**Region**	**x**	**y**	**z**
DMN	Precuneus	0	−55	26
DMN	IPL left	48	−63	30
DMN	IPL right	−40	−59	30
PF left	aPFC left	44	−55	42
PF left	IPL left	48	33	18
PF right	aPFC right	−28	−63	38
PF right	IPL right	−44	17	22
SM left	M1 left	36	−19	62
SM left	Cbl ant right	−24	−51	−30
SM left	Cbl post right	−24	−55	−58
SM left	M1 right	−36	−19	62
SM left	Cbl ant left	24	−51	−30
SM left	Cbl post left	24	−55	−58
SM right	S1 right	40	−31	42
SM right	S1 left	−40	−31	42
Cbl	Cbl left	24	−67	−38
Cbl	Cbl right	−24	−67	−38
Vis	V1 left	−20	−91	−6
Vis	V1 right	20	−91	−6
Visual Ext.	V5 right	44	−59	−18
Visual Ext.	V5 left	−40	−67	−10
Aud left	A1 left	64	−7	−2
Aud right	A1 right	−56	−11	2
Thal	Thal right	−24	9	−6
Thal	Thal left	28	13	−6

*Table of point locations used for seed-based functional connectivity analysis. The network name (i.e., independent component), anatomical region, and MNI coordinates are provided for each seed. Abbreviations: (DMN, default-mode network; PF left, left parieto-frontal network; PF right, right parietofrontal network; SM left, left sensorimotor network; Cbl, Cerebellum network; Vis, primary visual network; Visual Ext., extrastriate visual network; Aud left, left primary auditory network; Aud right, right primary auditory network; Thal, thalamus network). Region abbreviations: (IPL, inferior parietal lobule; aPFC, anterior prefrontal cortex; M1, primary motor cortex; S1, primary sensory cortex; V1, primary visual cortex; A1, primary auditory cortex; V5, middle temporal visual area)*.

### Statistical Analyses

#### Voxel-Based Analysis of BOLD Activation Maps and Spatial Network Maps

All spatial map group comparisons were performed in MNI space and corrected for multiple comparisons using FDR correction. Group differences in functional connectivity maps were calculated in a 4 mm resolution MNI space as output by MELODIC. In our AFNI pipeline, we upsampled the BOLD activation maps into 1 mm MNI space before group comparison. Three BOLD activation maps from AFNI and four spatial network maps from the FSL dual regression analysis were compared between groups and conditions by using voxel-based Student's *t*-tests. Since we were focused on sensorimotor function in this study, only the spatial maps of networks included in Table 2 were analyzed. Voxel-level Student's *t*-tests were performed to test contrasts between stroke and control groups. In the functional network maps, additional paired *t*-tests were performed to determine within-group contrasts between resting-state and search. Spatial clusters of significantly different voxels (*p* < 0.01) were identified. In order to account for multiple comparisons, a corrected alpha value of 0.05 was used to remove clusters less than a threshold size determined by 3dClustSim tool in AFNI. 3dClustSim, which corrects for multiple comparisons using FDR correction, was applied to both 4 mm and 1 mm MNI space templates to estimate cluster size thresholds for FDR correction. The cluster size threshold was 359 voxels for the 1 mm BOLD contrasts from AFNI and 8 voxels for the 4 mm functional connectivity maps from MELODIC. Multiple comparisons correction was further applied for the number of contrasts performed (3 activation maps and 20 functional networks). For between-network connectivity, a Student's *t*-test with was performed between stroke participants and age-matched controls for the edge strength between each pair of seed points. Multiple comparisons FWE correction was applied for the number of pairwise *t*-tests, which was (n^*^(n-1)/2) = (27^*^26/2) = 702.

#### Correlational Analysis With Clinical Functional Scores

We hypothesized that stroke-related differences in task-related BOLD activation and functional network spatial maps would be correlated with the clinical evaluations of sensorimotor impairment/function. A linear regression analysis was performed with each BOLD activation contrast and functional network maps as a predictor of the Box and Blocks Score for the impaired arm. No linear regressions were performed with our activation and connectivity measures and the Fugl-Meyer or the Wolf Motor scores because there was a ceiling effect in our stroke participants with the Fugl-Meyer upper extremity (Table 1), and the Wolf Motor correlated with the Fugl-Meyer (R = 0.87). The independent components that were compared between groups included the left and right sensorimotor networks. The *p*-values were multiplied by the number of analyzed networks to correct for multiple comparisons.

## Results

### The Search Task Produced Cortical Activation Patterns Within Motor and Multisensory Integration Areas

**Figure 2:**
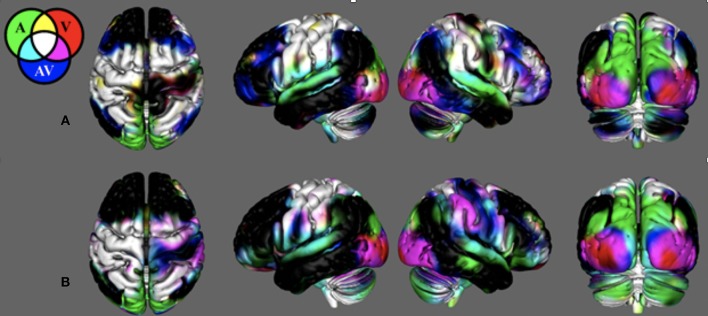
Search-task BOLD activation maps in healthy individuals. These activations were produced from the novel search task paradigm. Mean BOLD activation for the auditory (A_M_), visual (V_M_), and audiovisual (AV_M_) task conditions in **(A)** the young healthy adult group and **(B)** the control group age-matched to the stroke survivors. Note the BOLD contrasts are shown for the movement regressors.

Figure 2 demonstrates that in young healthy individuals and in the age-matched control group, our search task successfully produced unique cortical activation patterns for different sensory feedback conditions. Activity is reported where the group mean is significantly positive (*t* > 2.79, *p* < 0.01, corrected). Search task-related activation common to all conditions was detected in contralateral sensorimotor cortex, bilateral premotor cortex, bilateral somatosensory association cortex, and bilateral anterior cerebellum. Purely visual or auditory activity was found in the primary visual and auditory cortices. An inferior-to-superior spatial gradient in overlapping activation maps were seen along the bilateral occipital surface. The superior occipital gyrus activity was exclusive to the unimodal auditory feedback condition. The middle occipital gyrus responded to the auditory and audiovisual conditions.

In young adults, there was unique activation during the audiovisual feedback condition within the bilateral dorsolateral prefrontal cortex and bilateral posterior parietal cortex, corresponding to the anterior and posterior multimodal association areas. BOLD activation in the control group that was age-matched to the stroke survivors was similar with the young healthy adults. However, this group did not have unique activation within the prefrontal and posterior parietal areas. Figure S3 illiterates stroke activity and the difference between stroke and control.

### During Sensory-Guided Movement, BOLD Activation in Stroke Survivors Depends on Sensory Feedback Modality

**Figure 3:**
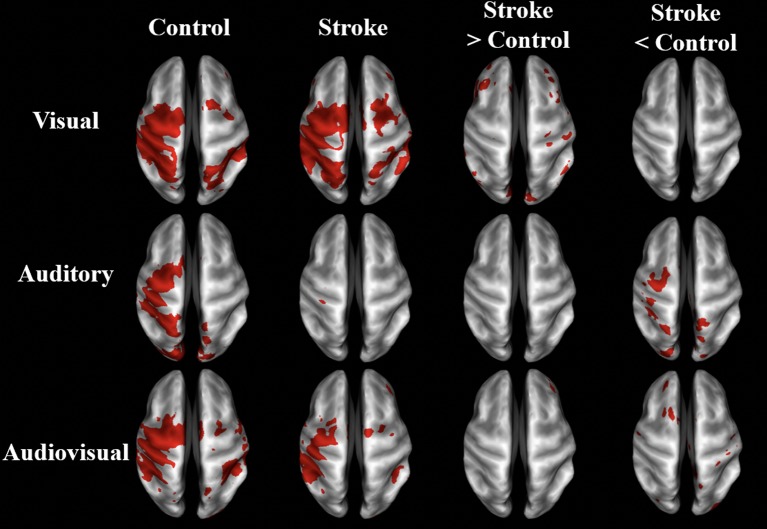
BOLD activation maps of stroke vs. controls. BOLD activation in visual, auditory, and audiovisual sensory guided movement. Group-averaged activity and significant differences (*p* < 0.01, un-corrected) are shown in stroke survivors (*n* = 10) and controls (*n* = 9) on inflated brain surfaces. The three sensory feedback conditions had similar activation in the contralateral sensorimotor cortex, bilateral premotor cortex, and bilateral somatosensory association cortex. Stroke participants had greater contralesional activation than controls during the visual condition and less ipsilesional activity during the auditory condition. The contralateral (ipsilesional) side is depicted on the left, and the ipsilateral (contralesional) side is depicted on the right.

As demonstrated in Figure 3 and Table 3, the BOLD activation in stroke survivors was dependent on the modality of sensory feedback. First, in the visual search condition, stroke survivors had similar activation to the age-matched controls within the active contralateral sensorimotor cortex. There was an increased activation in the contralesional prefrontal, posterior parietal, and sensorimotor cortices (*p* < 0.01, corrected). Increased activation was also observed within the ipsilesional prefrontal cortex (*p* < 0.01, corrected). Second, overall BOLD activity in stroke participants was lower than controls during the auditory feedback condition. Thirdly, the task-related BOLD activity in stroke survivors during the audiovisual search condition was significantly different from the age-matched controls in the contralesional inferior occipital gyrus and the posterior thalamic radiation. During the visual and the audiovisual search, contralesional regions positively correlated with the Box and Blocks score, with the exception of the superior temporal gyrus (Table 3). In the visual search task, the contralesional middle occipital gyrus, and superior temporal gyrus correlated with the Box and Blocks score; similarly, the inferior occipital and the posterior thalamic radiation correlated with the Box and Blocks score during the audiovisual task. As mentioned, the audio search task had decreased BOLD activity in the stroke group as compared to the controls, and these bilateral decreases were positively correlated with the Box and Blocks score.

**Table 3:** Localized group differences in BOLD activation.

**Condition**	**ROI**	**x**	**y**	**z**	**nVox**	**t**		**p (t)**	**corr B&B**		**p (slope)**
Visual	**MOG_R**	**46**	**−73**	**2**	**850**	**−3.16**	**^††^**	**0.00636**	**0.716**	**^†^**	**0.03610**
Search	**STG_R**	**68**	**−17**	**2**	**655**	**3.49**	**^††^**	**0.00321**	**−0.765**	**^†^**	**0.01972**
	SPG_R	31	−62	63	35	−2.00		0.06249	0.851	^††^	0.00389
Visual	Cbl_R	10	−46	−59	12	−2.35	^†^	0.03182	0.697	^†^	0.04527
Target	AG_L	−40	−75	49	96	2.34	^†^	0.03275	−0.845	^††^	0.00471
Auditory	**LG_L**	**−19**	**−82**	**−9**	**3616**	**−3.65**	**^††^**	**0.00221**	**0.844**	**^††^**	**0.00481**
Search	**LG_R**	**11**	**−80**	**−1**	**1234**	**−3.79**	**^††^**	**0.00167**	**0.698**	**^†^**	**0.04486**
	SPG_L	−27	−55	67	80	−2.73	^†^	0.01503	0.761	^†^	0.02112
	MCP_L	−12	−22	−33	50	−3.15	^††^	0.00651	0.672		0.05666
	STG_R	30	24	−34	12	−2.00		0.06236	0.742	^†^	0.02676
	SPG_L	−15	−63	71	12	−4.27	^††^	0.00062	0.655		0.06525
Auditory	**STWM_L**	**−52**	**−21**	**−1**	**6027**	**−3.77**	**^††^**	**0.00174**	**0.887**	**^††^**	**0.00154**
Target	**Cu_L**	**−4**	**−96**	**6**	**963**	**−2.58**	**^†^**	**0.02122**	**0.916**	**^††^**	**0.00049**
	**PoCWM_R**	**27**	**−35**	**74**	**528**	**−3.58**	**^††^**	**0.00265**	**0.837**	**^††^**	**0.00566**
	Cbl_R	8	−82	−47	296	−5.21	^††^	0.00009	0.698	^†^	0.04485
	IFG_R	35	17	14	166	−3.77	^††^	0.00173	0.632		0.08274
	PrCG_L	−58	−1	24	86	−2.44	^†^	0.02772	0.848	^††^	0.00427
	PrCG_L	−3	−30	74	37	−3.24	^††^	0.00546	0.885	^††^	0.00162
	STG_R	70	−22	−2	35	−3.63	^††^	0.00235	0.590		0.11181
	MCP_R	21	−64	−35	34	−3.79	^††^	0.00168	0.485		0.21533
Audiovisual	**IOG_R**	**39**	**−77**	**3**	**643**	**−4.46**	**^††^**	**0.00041**	**0.740**	**^†^**	**0.02721**
Search	**PTR_R**	**36**	**−61**	**0**	**484**	**−3.89**	**^††^**	**0.00138**	**0.807**	**^†^**	**0.01023**
Audiovisual	MTWM_L	−49	−49	1	70	4.77	^††^	0.00022	−0.602		0.10391
Target	Cbl_L	−31	−88	−29	52	−1.54		0.14873	0.910	^††^	0.00067
	ITG_L	−63	−53	−18	40	−3.55	^††^	0.00282	0.712	^†^	0.03807
	STG_L	−47	−41	5	32	2.93	^†^	0.01033	−0.692	^†^	0.04777
	PoCWM_L	−39	−27	36	25	2.46	^†^	0.02672	−0.752	^†^	0.02369
	SPG_L	−31	−49	34	12	2.48	^†^	0.02587	−0.743	^†^	0.02650

*Clusters surviving FDR correction are marked in bold font. Note that “corr B&B” is the Pearson correlation coefficient between the BOLD activation and Box and Blocks score in stroke participants. Also note, “Search” in this table refers to the A_M_, V_M_, and AV_M_ regressors and “Target” refers to the A_S_, V_S_, and AV_S_ regressors described in Section General linear model and cortical activation maps. ROI Acronyms: IFG, inferior frontal gyrus; IOG, inferior occipital gyrus; MOG, middle occipital gyrus; STG, superior temporal gyrus; SPG, superior parietal gyrus; ITG, inferior temporal gyrus; SMG, supramarginal gyrus; LG, lingual gyrus; Cbl, cerebellum; PoCG, postcentral gyrus; PrCG, precentral gyrus; AG, angular gyrus; Fu, fusiform gyrus; MTG, middle temporal gyrus; MCP, middle cerebellar peduncle; PTR, posterior thalamic radiation*.

† *p < 0.05*,

†† *p <0.01*.

### Stroke Participants Have Increased Contralesional Within-Network Functional Connectivity During the “Search” Task

**Figure 4:**
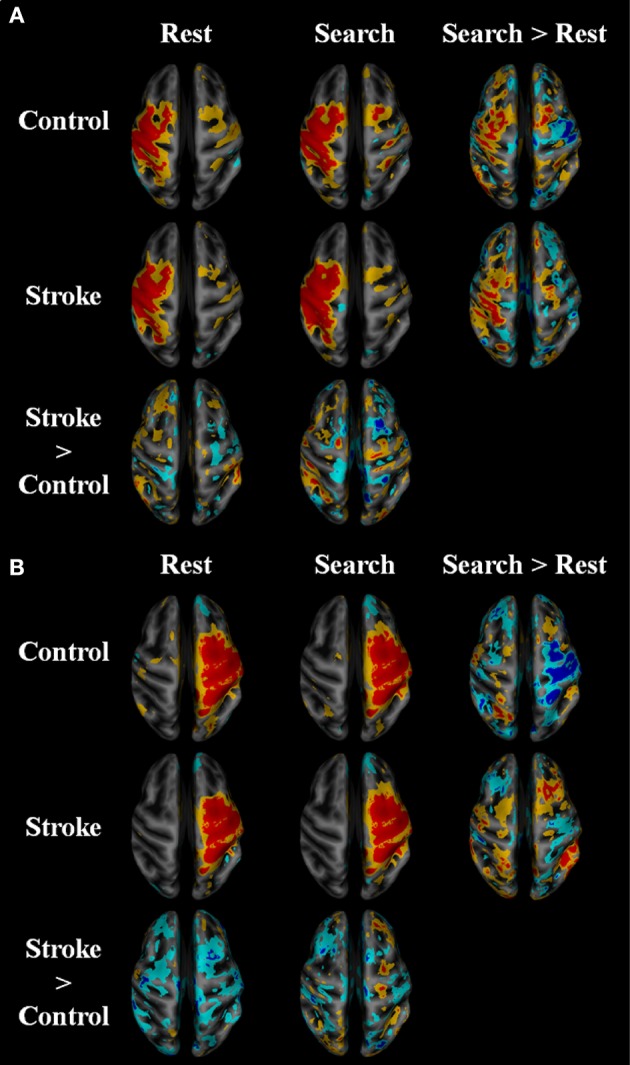
Within network functional connectivity: stroke vs. control functional network maps. Independent component spatial maps for the ipsilesional/contralateral **(A)** and contralesional/ipsilateral **(B)** sensorimotor networks. Stroke and control *t*-value group averages and differences for rest and task conditions are overlaid on an inflated cortical map surface. Group averages with a mean normalized component intensity above three are colored red. Difference maps show clusters >11 cubic millimeters after thresholding at *p* < 0.01. The ipsilesional side is depicted on the left, contralesional on the right. The colors represent *p*-values corresponding to the *t*-test run after the individual ICA maps were created with dual regression. Yellow and cyan are *p* < 0.05, where red and blue are *p* < 0.01. Blue/cyan represent significantly lower voxels and red/yellow represent significantly higher voxels for the test indicated.

Within-network functional connectivity information provided by the independent component analysis is shown in Figure 4; Table 4. Figure 4A presents the network that had the highest temporal correlation with the recorded movement. This network included the contralateral sensorimotor cortex, bilateral premotor cortex, and ipsilateral cerebellum, which had the highest temporal correlation with the modeled BOLD response (R = 0.716 across all participants). During both rest and the search task, this movement-related network was similar between stroke participants and age-matched controls. In controls, the participation of sensorimotor cortices became lateralized during the “search” task. Both hemispheres contributed to this shift in laterality, with greater positivity in contralateral sensorimotor areas and negative coefficients in the ipsilateral sensorimotor cortex. There was also an increased bilateral contribution from the supplementary motor area. These task-related network changes were not seen in stroke participants. Rather, contralateral somatosensory cortex and ipsilateral SMA and ventromedial premotor cortex increased in network participation. Thus, stroke participants had greater within-network participation in the ipsilateral hemisphere during the search task. This result also held after subtracting the resting-state network values.

**Table 4:** Localized group differences in network spatial maps.

**Run**	**IC**	**ROI**	**x**	**y**	**z**	**nVox**	**t**		**p (t)**	**corr B&B**		**p (slope)**
Rest	Right PF	ITG_R	44	−1	−44	13	−3.42	^††^	0.00360	0.928	^††^	0.00028
	DAN/CblL	SMG_R	68	−33	20	11	3.72	^††^	0.00188	−0.794	^†^	0.01276
											^††^	
Search	V1 Medial	LWM_R	12	−81	0	32	−3.18	^††^	0.00617	0.853	^††^	0.00377
	Left SM	Cbl_R	44	−65	−40	54	3.71	^††^	0.00192	−0.915	^††^	0.00049
	Left SM	Cbl_R	32	−53	−24	39	−3.26	^††^	0.00515	0.883	^††^	0.00171
	Left SM	PoCWM_L	−20	−33	40	35	3.52	^††^	0.00301	−0.857	^††^	0.00347
	Left SM	PrCG_R	36	−5	60	17	−4.43	^††^	0.00043	0.899	^††^	0.00097
	Right PF	AWM_R	36	−53	32	11	2.99	^††^	0.00900	−0.732	^†^	0.02957
											^††^	
S xor M	Right Insula	Fu_R	28	−81	−8	12	2.64	^†^	0.01955	−0.953	^††^	0.00020
	DMNmpf	MTG_L	−60	−21	−16	11	−4.28	^††^	0.00069	0.892	^††^	0.00305
	Left SM	Cbl_R	50	−61	−44	22	3.12	^††^	0.00720	−0.965	^††^	0.00007
	Left SM	Cbl_R	36	−53	−28	17	−3.01	^††^	0.00903	0.810	^†^	0.01711
	Central M1	Cbl_R	36	−77	−40	11	−3.81	^††^	0.00177	0.913	^††^	0.00152

† *p < 0.05*,

†† *p < 0.01*.

*MNI spatial coordinates are provided for clusters with significant group differences between stroke participants and age-matched controls*.

*ROI Acronyms: ITG, inferior temporal gyrus; SMG, supramarginal gyrus; LWM, lingual gyrus; Cbl, cerebellum; PoCWM, postcentral gyrus; PrCG, precentral gyrus; AWM, angular gyrus; Fu, fusiform gyrus; MTG, middle temporal gyrus*.

*IC Acronyms: PF, parietofrontal control network; DAN, dorsal attention network; V1 medial, central visual network; SM, sensorimotor network; DMNmpf, default-mode network medial prefrontal node*.

### Stroke Survivors Have Decreased Between-Network Interhemispheric Connectivity and Increased Functional Connectivity to Visual Areas

**Figure 5:**
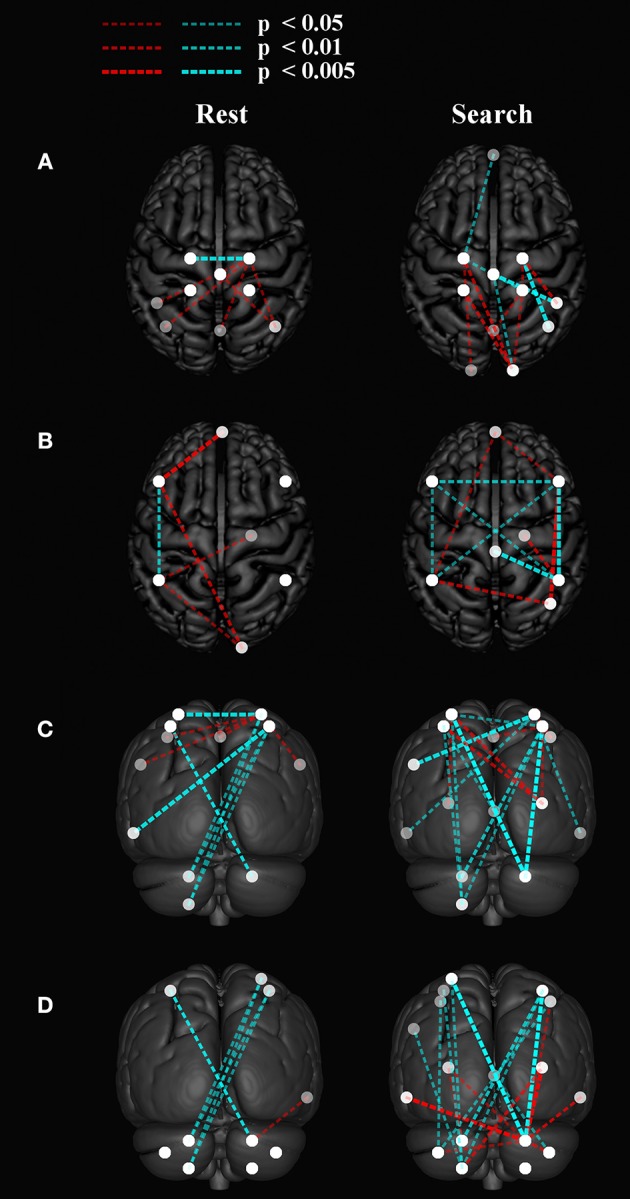
Group differences in between network functional connectivity. An inflated pial surface of a template brain is shown with color-coded independent components and functional network graph overlay. The ipsilesional side is depicted on the left, contralesional on the right. Stroke-related increased connections are in red and decreases are in cyan. The overhead view in **(A)** shows the connectivity to the left and right sensorimotor network nodes and in **(B)** presents connectivity to the bilateral parietofrontal networks. **(C)** The posterior views show connectivity differences to four **(C)** sensorimotor network nodes, and **(D)** eight cerebellar nodes. Note that all nodes are within brain tissue, although some of the network edges are difficult to visualize.

Between-network functional connectivity at rest and during the search task are shown in Figure 5. The resting-state functional connectivity between left and right sensorimotor networks was lower (*p* < 0.05, corrected) in the stroke group. During both the task and resting state, functional connectivity in stroke participants showed an increase in connectivity between the sensorimotor areas and the visual areas in the occipital lobe as shown in Figure 5A. During the “search” task the left and right parietofrontal networks demonstrated lower between-network functional connectivity, both between the parietofrontal areas and the rest of the networks. Connections to the cerebellum (Figure 5D) were also lower in participants with stroke.

### Decreased Task-Based Functional Connectivity With the Cerebellum During Sensorimotor Integration Correlates With Motor Impairment After Stroke

**Figure 6:**
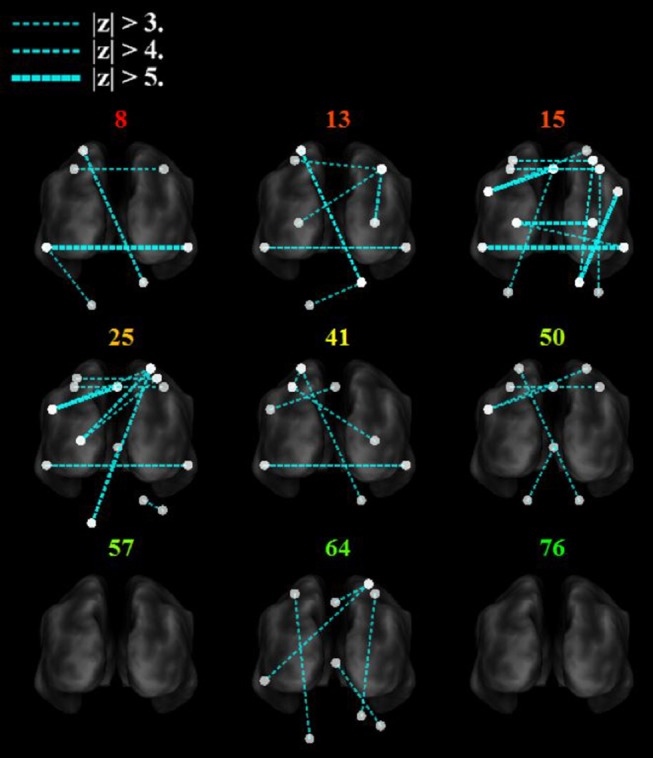
Functional trends in seed-based functional connectivity. Differences in functional connectivity in individual stroke survivors during the search task vary with Box and Blocks Score (colored number indicated on top of each brain). Note that the *z*-score is calculated as the difference in connectivity strength between the age-matched controls and the individual stroke participant. The ipsilesional side is depicted on the left, contralesional on the right. Note that all nodes are within brain tissue, although some areas in the cerebellum are difficult to visualize with this slice.

**Figure 7:**
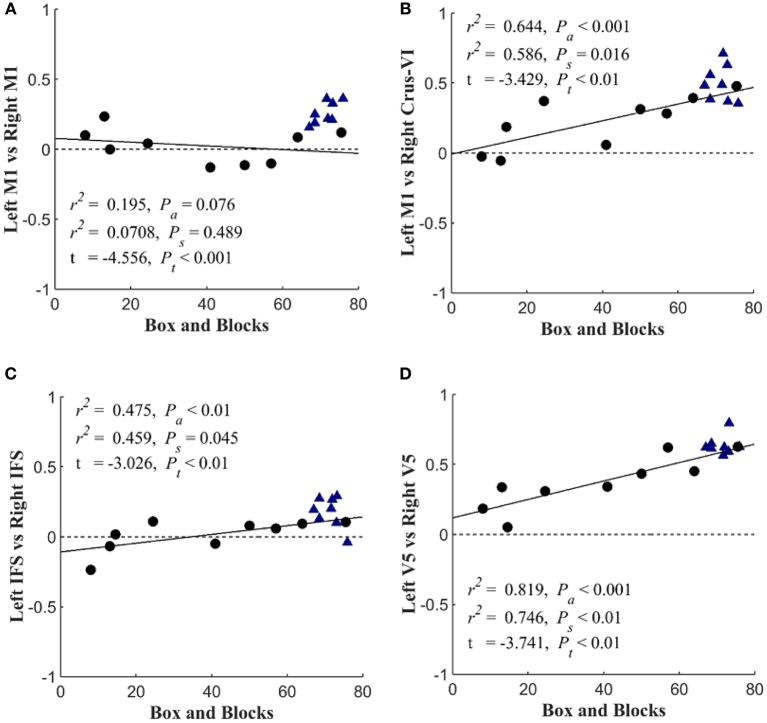
Scatterplots of seed-based connectivity during “search” task vs. motor function. Stroke participants are shown with black dots and controls are shown with blue triangles. The dependent variable in each plot is the partial correlation between two specified seed points. Linear regressions were performed within stroke participants and repeated across all participants (assuming that controls scored a 76). The first *r*^2^ and P_a_ are the coefficient of variation and slope's *p*-value for the regression analysis across all participants. The second *r*^2^ and P_s_ are associated with the analysis that included only stroke survivors. Group differences are reported by *t*-value and corresponding *P*_t_-value. The figure shows the Box and blocks correlation with **(A)** Left M1 & Right M1 connectivity, **(B)** Left M1 & Right Crus-V1 connectivity, **(C)** Left IFS & Right IFS connectivity, and **(D)** Left V5 & Right V5 connectivity.

Figure 6 shows that with the “search” task, decreases in network functional connectivity of the cerebellum and visual association areas were associated with Box and Blocks score in individuals with stroke. In stroke participants, the contralesional cerebellum had decreased functional connectivity with the active sensorimotor cortex as previously mentioned (Figure 5; *p* < 0.005, corrected). As shown in Figure 7, this cortico-cerebellar connectivity was significantly and positively correlated with Box and Blocks score (*r*^2^ = 0.64). Also, the connectivity of right and left extrastriate cortical regions (V5) measured during the search task were significantly and positively correlated with Box and Blocks score (*r*^2^ = 0.82). There were no significant correlations between resting state functional connectivity and the Box and Blocks score.

## Discussion

This study provides several novel findings that advance our understanding of network function of the brain after stroke. Consistent with our hypothesis, we found that the search task activated different parts of the brain dependent on visual, auditory, or audiovisual cues. For instance, in order to complete the visual search task control participants used greater contralateral activation, while stroke participants used greater ipsilateral activation. The connectivity analysis of the search task revealed more stroke-related deficits as compared to resting state. Of particular interest, we found a stroke-related increase in functional connectivity between the sensorimotor and visual areas only during the search task (not resting state), suggesting stroke survivors might rely more on visual feedback for motor control. The dependence of hand function on cerebellar and sensorimotor connectivity was only detected during the search task, indicative of the importance of performing task-based functional connectivity.

### Multisensory Control in Wrist Movement Evokes Activation in Bilateral Motor and Association Areas

The activation patterns seen in the neurologically intact control group revealed that wrist movement during single and multisensory search tasks elicits activation patterns mainly in the contralateral sensorimotor, and the bilateral premotor and somatosensory association cortices (Figure 2). As expected, the visual feedback task produced additional activation in the occipital lobe (44–46), while the auditory feedback produced activation in the temporal and superior occipital gyrus (47–49). Interestingly, the combined audiovisual condition in young adults also recruited areas in the dorsolateral prefrontal cortex and the bilateral posterior parietal cortex. Similarly, the within-network and between-network functional connectivity also suggests that the complex search task used in the current study connects motor and association areas bilaterally, which contrasts to the lateralized contralateral activation seen in finger tapping fMRI protocols (50, 51). This is notable in Figure 4A, where the independent component network with the highest temporal correlation with the recorded movement during the search tasks included the contralateral sensorimotor cortex, the bilateral premotor cortex, and the ipsilateral cerebellum (not visible in figure). Our study shows that a search task paradigm reveals the effects of multimodal sensory feedback on brain functional networks in neurologically intact participants, providing evidence that single-joint movement during multisensory control recruits areas of the brain beyond contralateral motor regions.

Brain activation was sensitive to the search task fMRI paradigm in stroke participants. As seen in Figure 3, stroke participants' task-related activation was dependent on the type of sensory feedback. Stroke participants had increased activation in the bilateral prefrontal cortex, and contralesional posterior parietal and sensorimotor cortices during the visual search condition. During auditory feedback, stroke participants had reduced ipsilesional activity. In the combined audiovisual search task, activation of the inferior occipital and the posterior temporal gyri was significantly reduced in stroke, and activation of these regions positively correlated with the Box and Blocks score (Table 3). These results suggest that task-related activation is sensory-dependent, and that multisensory integration is impacted by stroke (52).

Functional connectivity was also dependent on the search task in the current study. The within-network functional connectivity results suggest that stroke participants engage bilateral motor regions to a higher extent to complete the search task. Figure 4 shows that the functional connectivity within the ipsilateral sensorimotor network was greater during the search task as compared to rest in stroke participants. Whereas, the sensorimotor cortices in the neurologically intact group became more lateralized toward the contralateral hemisphere, the stroke group had greater within network participation in the ipsilateral hemisphere during the search task, suggesting that stroke participants engage bilateral motor networks during a search task and wrist movement. This observation is consistent with prior reports on brain activation (10, 53, 54).

### Task-Based Functional Connectivity Provides Unique Information About Sensorimotor Integration and Motor Control After Stroke

Consistent with our hypothesis, the search task revealed “local specialization” as evidenced by increased within-network functional connectivity, and greater between-network connectivity after stroke. The control group saw an increase in within-network connectivity in the contralateral sensorimotor areas and bilateral SMA (Figure 4A). As shown in Figure 4B, cortical areas within the ipsilateral sensorimotor network were lower during the search task in control participants, further suggesting task-related inhibition of the ipsilateral cortex in healthy individuals and lateralization toward the contralateral hemisphere (22). The increase in bilateral SMA contributions to the sensorimotor network with the task might be due to the strong reciprocal connections between the left and right SM (55). In contrast, the stroke group saw increased within network connectivity in the ipsilateral SMA and ventromedial premotor areas during the search task (Figure 4B). Due to lesion location in the contralateral hemisphere, stroke participants were unlikely to be able to increase contralateral cortical participation in the SM network with the task. Thus, the stroke group appeared to produce local reorganization in the ipsilateral side.

The task-based fMRI revealed stroke-related network connectivity changes that positively correlated with motor impairment (Figure 7). The resting-state functional connectivity between left and right sensorimotor networks was lower (*p* < 0.05) in the stroke group, which is consistent with other reports of decreased interhemispheric connectivity after stroke (21); however, these changes were not correlated with the box and blocks scores (Figure 7A). During the “search” task, reduced functional connectivity between the left and right V5 was strongly correlated with the box and blocks score in stroke individuals (Figure 7D). Additionally, the functional connectivity between the middle temporal gyrus and parietofrontal nodes showed stroke-related increases (Figure 5). The middle temporal gyrus has been shown to play a role in processing motion-related visual information (56).

### Clinical Correlations During the Search Task Reveal Sensorimotor Integration Deficits in Stroke

A unique finding in this study is a stroke-related increase in functional connectivity between the sensorimotor and visual areas during a search task, suggesting that stroke survivors may rely on visual feedback for motor control. During the auditory feedback conditions, stroke participant's auditory cortex was less active (Table 3; Figure 3). In contrast, ipsilesional cortical activity was more widespread in stroke participants than controls during the visuomotor task. While auditory feedback has been shown to improve weight bearing and gait characteristics in stroke survivors (57), individuals with stroke have been shown to rely on visual feedback for posturing (58). Additionally, visual feedback during grip-force control increases activation in the visual cortex, premotor cortex, supplementary motor areas, and the ipsilateral cerebellum in stroke participants (59), and limb apraxia has been associated with a deficit in visuo-motor integration due to lesions in the fronto-parietal motor network (60). The findings suggest that visual information during a complex motor task is an important source of feedback in stroke survivors and highlights the importance of measuring brain activation and calculating between-network connectivity during task-state fMRI.

Although the results showed an effect during visual feedback that was not present during the auditory condition, it would be overreaching to conclude that stroke survivors are more sensitive to visual over auditory feedback during fine-motor control. We do not know the visual vs. auditory sensitivity in each subject, which is information that would require a psychophysical exam. Although we did not record the dB gain of the maximum auditory feedback, a reasonable volume was used during the experiment. The volume was set such that the subject reported that the auditory feedback could be heard.

In addition to identifying stroke participant's dependence on visual feedback for motor control, this work emphasized the importance of cerebellar functional connectivity after stroke, specifically during sensorimotor integration. As indicated in Figure 5, the bilateral cerebellum exhibited stroke-related decreases in functional connectivity during both at rest and during the search task. Additionally, the connectivity between the ipsilateral cerebellar crus VI and the contralateral M1 was positively correlated with the box and blocks score when tested using the search task (Figure 7B). Involvement of the cerebellum in different networks involving movement and multisensory integration give it a critical role in brain plasticity after stroke (61). The motor cortex and cerebellum together have been shown to be involved in plasticity during motor training and in sensorimotor integration (62). Past imaging studies have shown that the connectivity between the cerebellum and parietofrontal areas are related to post-stroke motor function (63, 64).

### Study Limitations

This study is limited by its small sample size for each group, especially for the age matched control and stroke participants. Head motion is a potential confounding factor in any study using functional activity or connectivity MRI, especially those involving task-based paradigms and patient populations. Indeed, head motion has been found in past studies to be greater in patients than in controls, and also increases with age (65). Van Dijk et al. found that although most variability in functional connectivity is not associated with head movement, there is significantly reduced functional coupling between the parietofrontal and default-mode network nodes in young adults. Furthermore, greater mean head motion leads to increased local functional coupling and interhemispheric connectivity between sensorimotor areas. Stroke participants in this study had significantly greater mean head motion than controls (*p* < 0.05) at rest and during the search task. Thus, our findings that stroke survivors had decreased between-network connectivity to the bilateral parietofrontal networks and increased within-network connectivity contralesionally could be in part due to increased head motion. However, we observed decreased interhemispheric connectivity, opposite to what was observed by Van Dijk et al. Since head motion was regressed out of the original data before our seed-based analysis, we do not believe that it was the prime contributor to our findings.

Changes in vasculature after stroke can lead to differences in neurovascular coupling near the lesion, which may influence correlations of these voxels with distant areas. Differences in brain structure can have an impact on functional connectivity metrics due to changes in the partial volume of gray matter (66). Using simulations, Dukart and Bertolino showed that between-group differences in brain structure leads to significant differences in functional connectivity between the groups. Due to the large variability in lesion location in this study, partial volume was not expected to have a significant impact on functional connectivity results.

Non-stroke related lateralization of cortical activation and functional connectivity is a potential confounding factor in this study. Studies of healthy adults have shown significant lateralization of resting-state functional connectivity (67). Nielson et al. showed that there are 20 “lateralization hubs” that have the most lateralized functional connectivity. Some of these hubs included the dorsolateral prefrontal cortex, supplementary motor area, premotor cortex, Broca's area, insula, and junctions between the parietal, occipital, and temporal lobes. Many of these regions are unimodal and multimodal sensory association areas. Handedness of our participants and the procedures of flipping the brain over the mid-sagittal plane to place all lesions on the same side of the brain could have had an impact on the results.

Spatial group differences in BOLD contrasts and functional connectivity could have been affected by group differences in the level of head motion during the task. The fsl_motion_outliers program in the FMRIB Software Library was used to estimate mean frame-wise displacement (FD) and mean square of successive differences (DVARS) (68) from the BOLD data during the search task runs of each subject. Shown in Figure S4, both metrics of head motion were significantly different (*p* < 0.01) between groups, but neither DVARS (r2 = 0.02) nor FD (r2 = 0.08) were correlated with Box and Blocks score. Although head motion regressors were included in all fMRI analyses in this study, greater motion in stroke subjects may have reduced the peak values of the functional connectivity maps.

Differences between study groups in range of wrist movement, and task performance could also impact the interpretation of our imaging findings. We report the number of completed search-task trials as a rough estimate of performance in Figure S5a. On average, stroke subjects completed 49.4 trials, which was significantly (*p* < 0.01) >61.1 trials completed in age-matched controls. However, the number of completed trials did not correlate (r2 = 0.04) with the Box and Blocks score. Shown in Figures S5a–c, the number of search trials (Figure S5a) and the average error accumulated (Figures S5b,c) was not correlated with the box and blocks scores. Shown in Figures S5d,e, the range of wrist motion was not significantly different between groups nor correlated with Box and Blocks score in stroke subjects.

## Conclusion

In conclusion, our novel functional MRI task paradigm involving wrist movement and multisensory feedback revealed changes in BOLD activation and functional connectivity after stroke, suggesting that task-based fMRI can highlight alterations in brain functional networks after stroke. We documented widespread bilateral activation in neurologically intact participants during an audiovisual search task, indicating that multisensory wrist control recruits primary motor and association areas. In contrast, stroke participant's activation patterns were task dependent; visual feedback produced increased contralesional activation compared to controls, while auditory feedback resulted in decreased activation ipsilesionally. Additionally, our within-network functional connectivity analysis detected a task-related lateralization to contralateral sensorimotor regions in control participants that was not found in stroke. Lastly, between-network functional connectivity during the search task revealed decreased connectivity between the ipsilesional sensorimotor cortex and the contralesional cerebellum, and between interhemispheric V5 regions, which both correlated with box and blocks scores of hand function. These results suggest that task-based functional connectivity provides detail on changes in brain networks in stroke survivors. The data also highlight the importance of cerebellar connections for recovery of arm function after stroke. Future work will investigate the role of structural connectivity in these mechanistic changes between sensorimotor and sensory integration networks.

## Data Availability

The raw data supporting the conclusions of this manuscript will be made available by the authors, without undue reservation, to any qualified researcher.

## Ethics Statement

This study was carried out in accordance with the recommendations of the Institutional Review Boards of the Medical College of Wisconsin and Marquette University with written informed consent from all subjects. All subjects gave written informed consent in accordance with the Declaration of Helsinki. The protocol was approved by the Institutional Review Board of the Medical College of Wisconsin.

## Author Contributions

BK involved in conception and design of work, software development, subject recruitment, data acquisition, image processing, results interpretation, manuscript drafting, and editing. KV and MS involved in results interpretation, manuscript drafting, and editing. AH involved in conception and design of work, clinical measures, results interpretation. BS involved in conception and design of work, results interpretation, manuscript drafting, and editing.

### Conflict of Interest Statement

The authors declare that the research was conducted in the absence of any commercial or financial relationships that could be construed as a potential conflict of interest.

## Supplementary Material

Figure S1 Stroke lesion distribution. Spatial distribution of lesioned voxels in the stroke subject cohort overlaid on eight slices in MNI space. Note that three stroke subjects' images have been flipped over the *x*-axis so that lesions are on the left and cerebellar lesions on the right. The lesions share limited overlap, with three lesions overlapping in the posterior limb of the internal capsule and two lesions overlapping in the superior temporal gyrus and sensorimotor cortex. Regions limited to a single lesion include the cerebellum, brainstem, and insula, and frontal gray and white matter.

Figure S2 Overlay of task-based network time-course and GLM model. A comparison of the active sensorimotor network time-course (blue) and the modeled BOLD response to the motor-only condition (dotted black line) for a single control participant.

Figure S3 BOLD Activation Maps for the search task. Respectively, the visual, auditory, and audiovisual feedback conditions are mapped to red, green, and blue. Cortical surface overlay with auditory, visual, and audiovisual brain activation regressors mapped to red, green, and blue by t-value. Coefficients are shown for **(a)** young adults, **(b)** age-matched controls, **(c)** stroke subjects, differences between groups are shown for Stroke > Controls **(d)** and Controls > Stroke **(e)**, where t > 2.3.

Figure S4 Head motion in stroke subjects and age-matched controls. Comparison of head motion across all search task trials in stroke subjects (filled circles) and age-matched controls (triangles). **(a)** Mean framewise displacement (FD) and **(b)** mean square of successive differences (DVARS) (68) are plotted against Box and Blocks score. Age-matched controls were given an artificial score of 76 for visualization purposes only. Correlations were only performed on stroke subjects.

Figure S5 Wrist range of motion and task performance in stroke subjects and age-matched controls. Comparison of stroke subjects (filled circles) and age-matched controls (triangles) in range of wrist motion and number of completed search task trials. **(a)** Number of search task trials, **(b)** Average θ_x_ error, **(c)** and θ_y_ error in radians, **(d)** angular range of θ_x_ dominantly controlled by supination/pronation, **(e)** angular range of θ_y_ associated with radial/ulnar deviation, plotted against Box, and Blocks score. Age-matched controls were given an artificial score of 76 for visualization purposes only. Correlations were only performed on stroke subjects.
