# Supplementary figures and images for: Tasked-Based Functional Brain Connectivity in Multisensory Control of Wrist Movement After Stroke
Source: Front Neurol. 2019 Jun 13;10:609. doi: 10.3389/fneur.2019.00609 (PMC6585311; doi:10.3389/fneur.2019.00609)

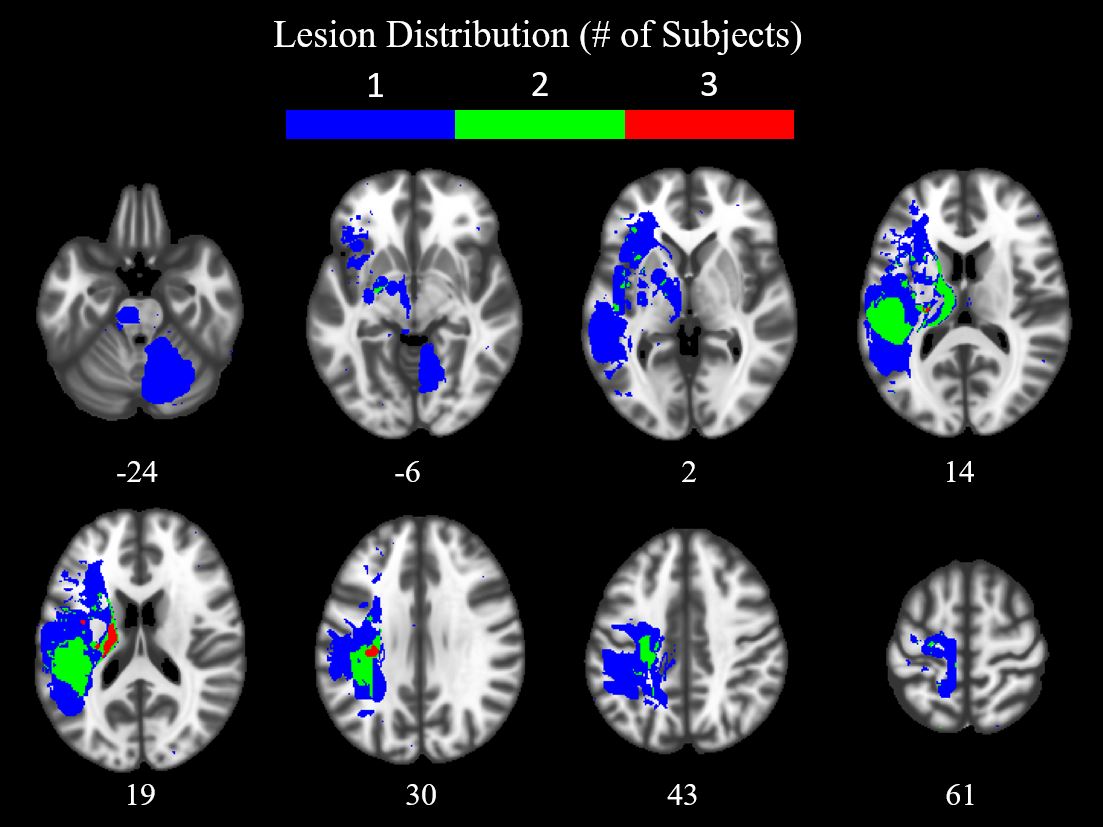

Supplement: Figure S1 — Stroke lesion distribution. Spatial distribution of lesioned voxels in the stroke subject cohort overlaid on eight slices in MNI space. Note that three stroke subjects' images have been flipped over the x-axis so that lesions are on the left and cerebellar lesions on the right. The lesions share limited overlap, with three lesions overlapping in the posterior limb of the internal capsule and two lesions overlapping in the superior temporal gyrus and sensorimotor cortex. Regions limited to a single lesion include the cerebellum, brainstem, and insula, and frontal gray and white matter. [file Image_1.JPEG]

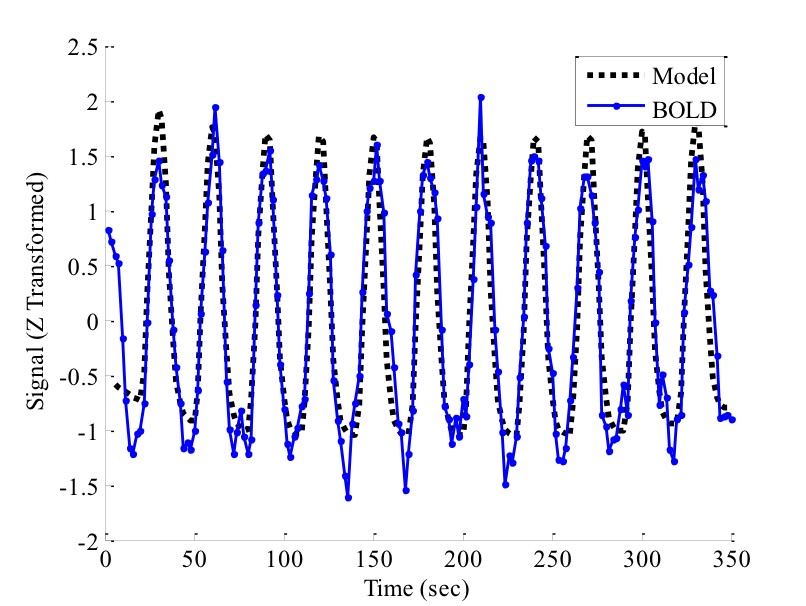

Supplement: Figure S2 — Overlay of task-based network time-course and GLM model. A comparison of the active sensorimotor network time-course (blue) and the modeled BOLD response to the motor-only condition (dotted black line) for a single control participant. [file Image_2.JPEG]

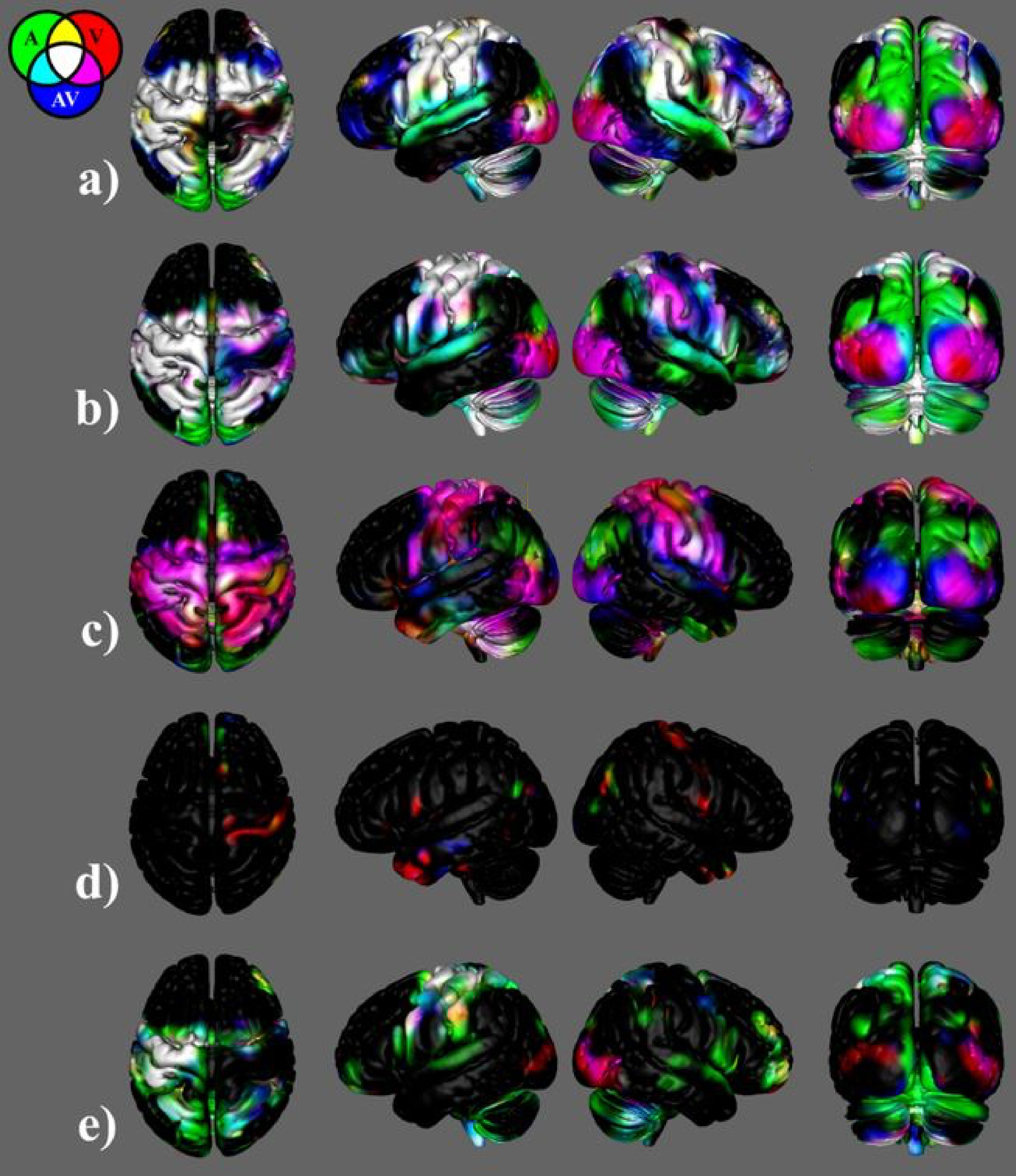

Supplement: Figure S3 — BOLD Activation Maps for the search task. Respectively, the visual, auditory, and audiovisual feedback conditions are mapped to red, green, and blue. Cortical surface overlay with auditory, visual, and audiovisual brain activation regressors mapped to red, green, and blue by t-value. Coefficients are shown for (a) young adults, (b) age-matched controls, (c) stroke subjects, differences between groups are shown for Stroke > Controls (d) and Controls > Stroke (e), where t > 2.3. [file Image_3.jpg]

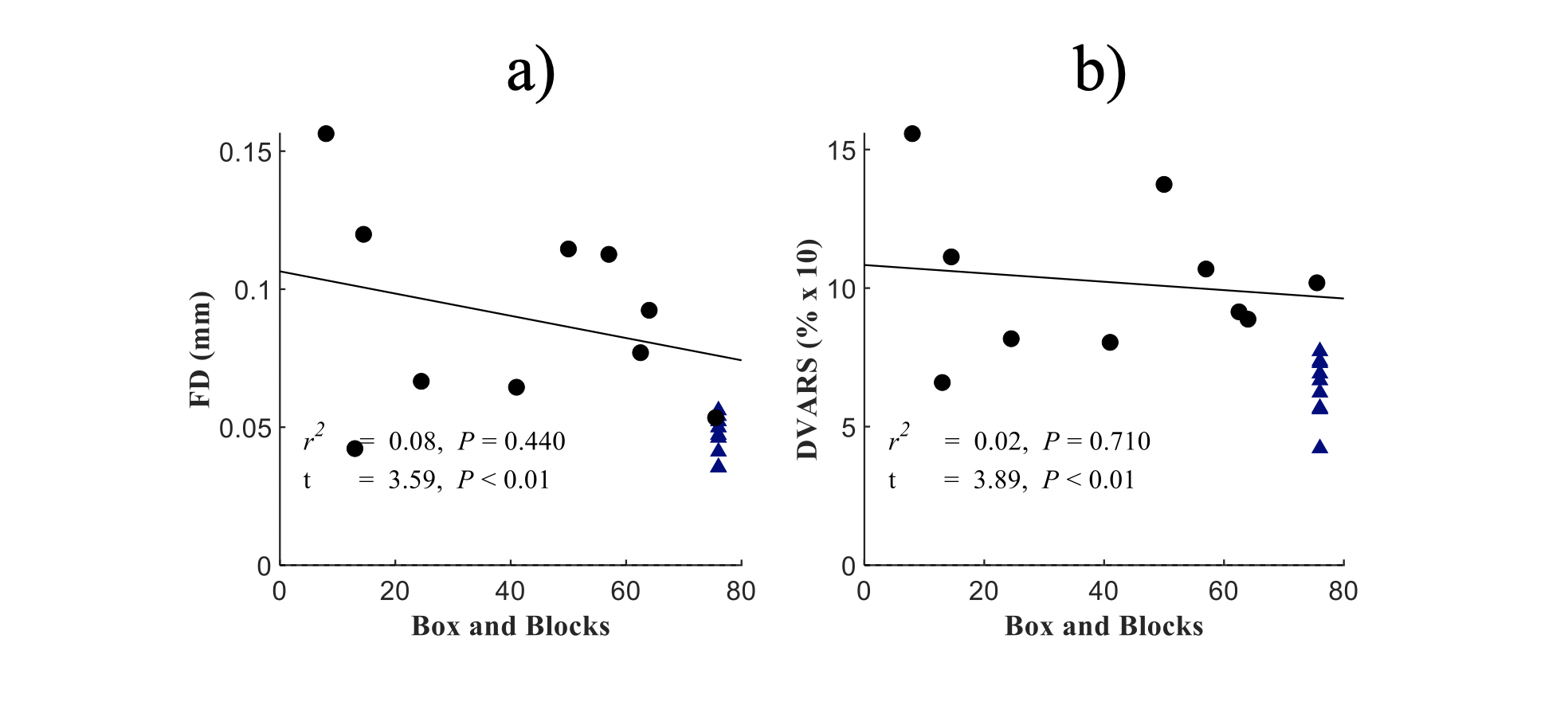

Supplement: Figure S4 — Head motion in stroke subjects and age-matched controls. Comparison of head motion across all search task trials in stroke subjects (filled circles) and age-matched controls (triangles). (a) Mean framewise displacement (FD) and (b) mean square of successive differences (DVARS) (68) are plotted against Box and Blocks score. Age-matched controls were given an artificial score of 76 for visualization purposes only. Correlations were only performed on stroke subjects. [file Image_4.JPEG]

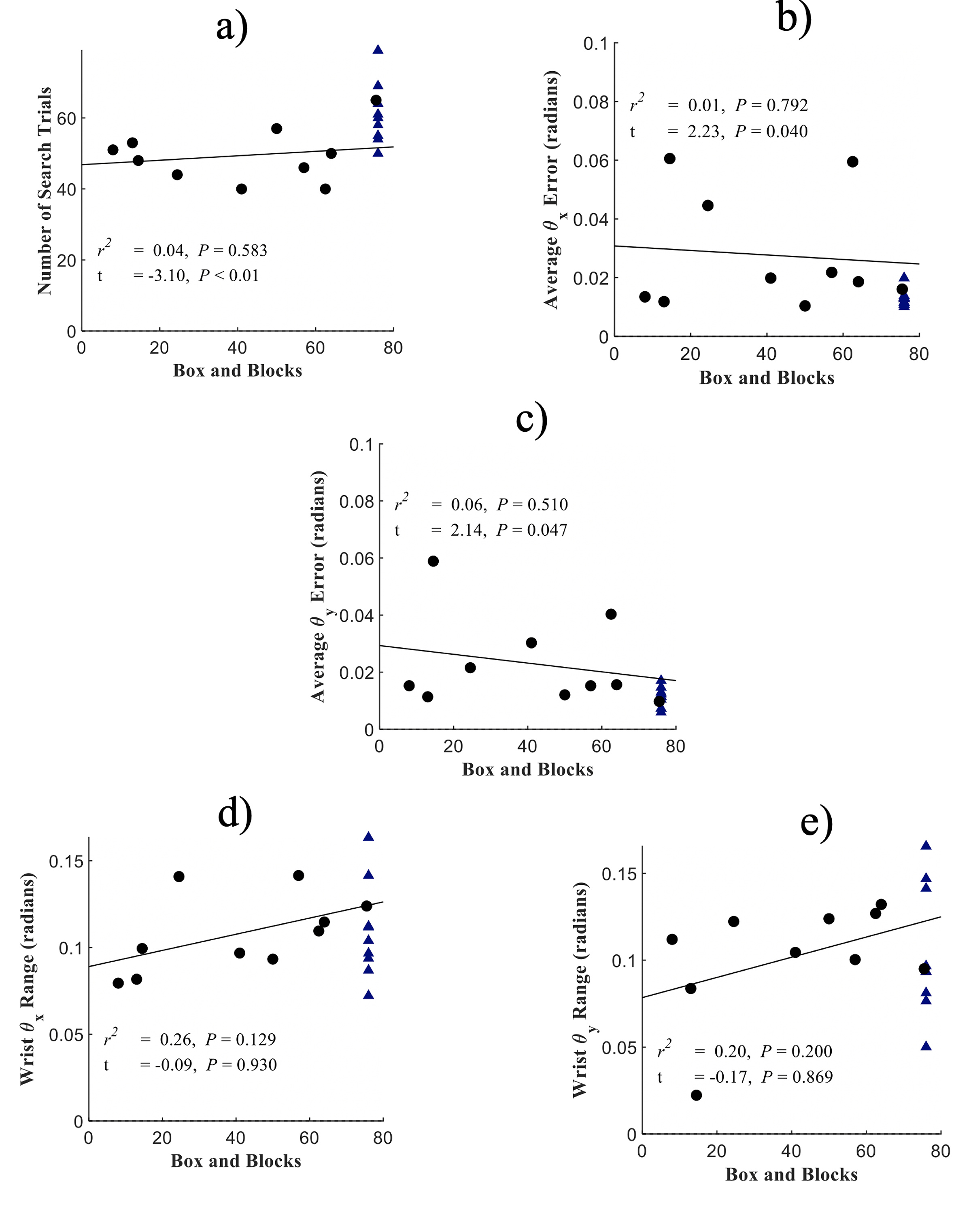

Supplement: Figure S5 — Wrist range of motion and task performance in stroke subjects and age-matched controls. Comparison of stroke subjects (filled circles) and age-matched controls (triangles) in range of wrist motion and number of completed search task trials. (a) Number of search task trials, (b) Average θx error, (c) and θy error in radians, (d) angular range of θx dominantly controlled by supination/pronation, (e) angular range of θy associated with radial/ulnar deviation, plotted against Box, and Blocks score. Age-matched controls were given an artificial score of 76 for visualization purposes only. Correlations were only performed on stroke subjects. [file Image_5.JPEG]
